# Supplementary material for: Hot spot prediction in protein-protein interactions by an ensemble system
Source: BMC Syst Biol. 2018 Dec 31;12(Suppl 9):132. doi: 10.1186/s12918-018-0665-8 (PMC6311905; doi:10.1186/s12918-018-0665-8)
Supplement: Supplementary file 2 — All relevant MATLAB code. A simple Matlab implement of our predictor. (ZIP 19,742 kb) [file 12918_2018_665_MOESM2_ESM.zip › Additional file 2/Read ASA/ASA website/SKEMPI/1EFNA.htm]

 NetSurfP - Protein Surface Accessibility and Secondary Structure Predictions

  

|  |  |  |
| --- | --- | --- |
|  |  | NetSurfP - Protein Surface Accessibility and Secondary Structure Predictions  Technical University of Denmark |

  


---

```
# For publication of results, please cite:
# A generic method for assignment of reliability scores applied to solvent accessibility predictions.
# Bent Petersen, Thomas Nordahl Petersen, Pernille Andersen, Morten Nielsen and Claus Lundegaard
# BMC Structural Biology 2009, 9:51 doi:10.1186/1472-6807-9-51
#
# Column 1: Class assignment - B for buried or E for Exposed - Threshold: 25% exposure, but not based on RSA
# Column 2: Amino acid
# Column 3: Sequence name
# Column 4: Amino acid number
# Column 5: Relative Surface Accessibility - RSA
# Column 6: Absolute Surface Accessibility
# Column 7: Z-fit score for RSA prediction
# Column 8: Probability for Alpha-Helix
# Column 9: Probability for Beta-strand
# Column 10: Probability for Coil
E A  1EFNA                  1    0.515  56.709  -0.356   0.005   0.045   0.951
B L  1EFNA                  2    0.156  28.527   0.385   0.001   0.959   0.040
B F  1EFNA                  3    0.071  14.169   0.476   0.000   0.983   0.017
B V  1EFNA                  4    0.154  23.731   1.430   0.000   0.983   0.017
B A  1EFNA                  5    0.033   3.670  -0.249   0.000   0.983   0.017
B L  1EFNA                  6    0.093  17.102   1.306   0.001   0.959   0.040
B Y  1EFNA                  7    0.152  32.547   0.961   0.003   0.718   0.279
B D  1EFNA                  8    0.279  40.189   0.299   0.005   0.336   0.660
B Y  1EFNA                  9    0.141  30.196   0.442   0.020   0.205   0.775
E E  1EFNA                 10    0.574 100.295   1.838   0.018   0.088   0.893
B A  1EFNA                 11    0.241  26.514  -0.838   0.018   0.047   0.935
E I  1EFNA                 12    0.657 121.471  -0.199   0.005   0.015   0.979
E T  1EFNA                 13    0.613  85.009   0.735   0.005   0.015   0.979
E E  1EFNA                 14    0.686 119.792   0.548   0.018   0.019   0.964
E D  1EFNA                 15    0.617  88.866  -0.796   0.018   0.047   0.935
E D  1EFNA                 16    0.314  45.262  -0.074   0.019   0.141   0.840
B L  1EFNA                 17    0.061  11.242   0.781   0.021   0.279   0.699
E S  1EFNA                 18    0.394  46.189   1.490   0.022   0.359   0.619
B F  1EFNA                 19    0.060  12.122   1.070   0.021   0.279   0.699
E H  1EFNA                 20    0.497  90.404   1.765   0.018   0.088   0.893
E K  1EFNA                 21    0.610 125.518   1.441   0.018   0.047   0.935
E G  1EFNA                 22    0.511  40.231  -0.647   0.005   0.015   0.979
B E  1EFNA                 23    0.151  26.380   0.240   0.004   0.197   0.799
E K  1EFNA                 24    0.281  57.699   1.930   0.001   0.900   0.099
B F  1EFNA                 25    0.036   7.185   0.856   0.001   0.959   0.040
B Q  1EFNA                 26    0.225  40.203   2.233   0.001   0.959   0.040
B I  1EFNA                 27    0.086  15.966   0.957   0.001   0.959   0.040
B L  1EFNA                 28    0.119  21.826   1.440   0.001   0.959   0.040
E N  1EFNA                 29    0.432  63.201   0.436   0.001   0.900   0.099
E S  1EFNA                 30    0.433  50.806   0.929   0.004   0.616   0.381
E S  1EFNA                 31    0.545  63.886   0.046   0.004   0.197   0.799
E E  1EFNA                 32    0.725 126.623  -0.330   0.005   0.045   0.951
E G  1EFNA                 33    0.769  60.528   0.200   0.003   0.003   0.994
E D  1EFNA                 34    0.326  46.948  -0.258   0.005   0.045   0.951
B W  1EFNA                 35    0.127  30.471   0.185   0.004   0.616   0.381
B W  1EFNA                 36    0.049  11.785   0.150   0.001   0.900   0.099
E E  1EFNA                 37    0.253  44.234   1.594   0.001   0.959   0.040
B A  1EFNA                 38    0.021   2.303  -0.762   0.001   0.959   0.040
E R  1EFNA                 39    0.344  78.684   0.946   0.001   0.959   0.040
B S  1EFNA                 40    0.186  21.787  -0.452   0.001   0.900   0.099
E L  1EFNA                 41    0.344  62.931  -0.295   0.004   0.420   0.576
E T  1EFNA                 42    0.540  74.884  -1.668   0.004   0.138   0.858
E T  1EFNA                 43    0.637  88.338  -0.201   0.005   0.045   0.951
E G  1EFNA                 44    0.462  36.320  -0.794   0.005   0.015   0.979
E E  1EFNA                 45    0.351  61.337   0.875   0.005   0.336   0.660
E T  1EFNA                 46    0.346  47.976   1.538   0.003   0.718   0.279
B G  1EFNA                 47    0.039   3.038  -1.582   0.002   0.816   0.182
B Y  1EFNA                 48    0.136  29.106   0.864   0.021   0.756   0.223
B I  1EFNA                 49    0.063  11.618   0.731   0.004   0.616   0.381
B P  1EFNA                 50    0.099  13.991   0.087   0.021   0.279   0.699
B S  1EFNA                 51    0.222  25.995  -0.700   0.058   0.017   0.925
B N  1EFNA                 52    0.356  52.060  -0.514   0.052   0.084   0.864
B Y  1EFNA                 53    0.182  38.829   0.525   0.069   0.386   0.545
B V  1EFNA                 54    0.108  16.615  -0.247   0.023   0.655   0.322
E A  1EFNA                 55    0.491  54.086   1.145   0.021   0.756   0.223
E P  1EFNA                 56    0.523  74.200   1.565   0.023   0.655   0.322
E V  1EFNA                 57    0.486  74.729  -0.520   0.005   0.015   0.979

Explain the output.  Go back.
```

---
